# The Fabrication and Characterization of InAlAs/InGaAs APDs Based on a Mesa-Structure with Polyimide Passivation

**DOI:** 10.3390/s19153399

**Published:** 2019-08-02

**Authors:** Jheng-Jie Liu, Wen-Jeng Ho, June-Yan Chen, Jian-Nan Lin, Chi-Jen Teng, Chia-Chun Yu, Yen-Chu Li, Ming-Jui Chang

**Affiliations:** 1 Department of Electro-Optical Engineering, National Taipei University of Technology, No. 1, Section 3, Zhongxial East Road, Taipei 10608, Taiwan; 2 Tyntek Corp., No. 15, Kejung Rd., Chunan Science Park, Chunan, Miaoli County 350, Taiwan

**Keywords:** avalanche photodiodes, InAlAs, InP, multiplication gain, polyamide passivation, eye-diagrams

## Abstract

This paper presents a novel front-illuminated InAlAs/InGaAs separate absorption, grading, field-control and multiplication (SAGFM) avalanche photodiodes (APDs) with a mesa-structure for high speed response. The electric fields in the InAlAs-multiplication layer and InGaAs-absorption layer enable high multiplication gain and high-speed response thanks to the thickness and concentration of the field-control and multiplication layers. A mesa active region of 45 micrometers was defined using a bromine-based isotropic wet etching solution. The side walls of the mesa were subjected to sulfur treatment before being coated with a thick polyimide layer to reduce current leakage, while lowering capacitance and increasing response speeds. The breakdown voltage (V_BR_) of the proposed SAGFM APDs was approximately 32 V. Under reverse bias of 0.9 V_BR_ at room temperature, the proposed device achieved dark current of 31.4 nA, capacitance of 0.19 pF and multiplication gain of 9.8. The 3-dB frequency response was 8.97 GHz and the gain-bandwidth product was 88 GHz. A rise time of 42.0 ps was derived from eye-diagrams at 0.9 V_BR_. There was notable absence of intersymbol-interference and the signals remained error-free at data-rates of up to 12.5 Gbps.

## 1. Introduction

Avalanche photodiodes (APDs) featuring a separate absorption and multiplication (SAM) layer-structure are widely used to produce low-noise optical detectors of high sensitivity [1,2]. InGaAs/InP APDs comprise an absorption layer of In_0.53_Ga_0.47_As (hereafter referred to as InGaAs) with a multiplication layer of InP. These devices have long been used as high-performance detectors in optical communication systems operating at wavelengths of 1310 or 1550 nm [3,4,5]. However, In_0.52_Al_0.48_As (hereafter referred to as InAlAs) is attracting considerable attention for use in the multiplication layer for three main reasons: (1) The electron/hole ionization coefficient of InAlAs is larger than that of InP [6,7,8,9]; (2) The electron mobility of InAlAs is greater than that of InP [10,11]; and (3) The ionization coefficient of InGaAs/InAlAs APDs is less sensitive than InGaAs/InP APDs to variations in temperature [6,7,8,9,12,13,14,15]. As a result, InGaAs/InAlAs APDs achieve far higher performance in terms of noise, gain-bandwidth, response time and resistance to temperature variation [14,16,17].

In this study, we fabricated front-side illuminated mesa-type InGaAs/InAlAs APDs based on a separate absorption, grading, field-control and multiplication (SAGFM) layer structure in conjunction with multi-step mesa etching and sulfur/polyimide passivation to achieve high-speed operations and high sensitivity [18,19,20,21,22]. Electric-field distribution in the multiplication and absorption layers is well controlled to enable high multiplication gain and low noise. The proposed device was evaluated in terms of dark current, breakdown voltage, multiplication gain, capacitance, incident optical power dynamic range, aging test, frequency response and transmission speeds at room temperature.

## 2. Experiments

### 2.1. Epitaxial Layer Design and the Calculation of Electric-Field Profiles

The proposed avalanche photodiode (APD) was configured using separate absorption, grading, field-control and multiplication (SAGFM) layer-structure. Table 1 details the parameters of the epitaxial layers, which were grown using molecular beam epitaxy (MBE) on semi-insulated InP wafers with (100) orientation and low etch pit density (EPD), measuring two inches in diameter and 350 μm in thickness. The quality of the epitaxial layers was verified via double-crystal X-ray diffraction (to check for lattice mismatch between hetero-junction layers less than 300 ppm), photoluminescence (to assess the optical properties of InGaAlAs, InAlAs, InGaAs and InP layers) and electrochemical capacitance-voltage (to measure the carrier concentrations of P^+^-InGaAs contact-layer, p-InP window-layer, i-InGaAs absorber, p-InAlAs field control-layer and n^+^-InAlAs contact-layer). Scanning electron microscopy (SEM) was used to measure the thickness of the epitaxial layers and characterize the interface between layers. Note that the doping concentration and thickness of the field control layer were factors of critical importance. All measurements pertaining to the epitaxial layers were confirmed by the semiconductor foundry (Intelligent Epitaxy Technology Inc., Richardson, TX, USA).

The electric field profiles of the multiplication and absorption layers were controlled by adjusting the doping and thickness of the p-InAlAs field-control layer, InAlAs multiplication layer and the InGaAs absorption layer. The electric field distribution was calculated using simulation software in accordance with the proposed layer parameters. The proposed SAGFM APD was fabricated using an un-doped InAlAs multiplication layer (0.3-μm-thick), an un-doped InGaAs absorption layer (1.2-μm-thick) and a p-InAlAs field-control layer (0.2-μm-thick) to enable high-speed operations.

### 2.2. Fabrication and Characterization of InAlAs/InGaAs APDs

**Figure 1:**
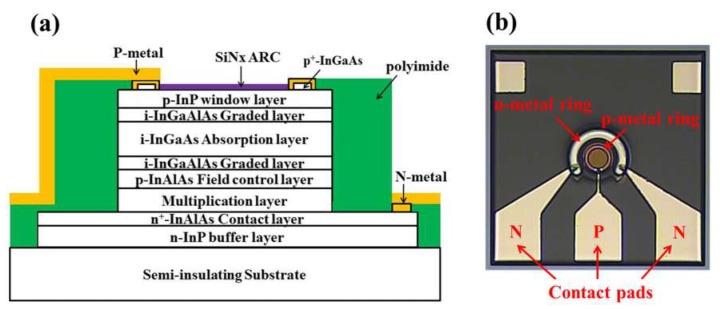
Schematic diagram showing InAlAs/InGaAs separate absorption, grading, field-control and multiplication (SAGFM) avalanche photodiode: (**a**) Side-view, (**b**) top-view.

Epitaxial wafers were cut into samples measuring 1 × 1 cm^2^ for the fabrication of SAGFM APD devices. After standard cleaning, a 5-μm-wide p^+^-InGaAs circular contact-ring (30-μm diameter) was created on the front surfaces of the samples through the selective etching of InGaAs from InP using a solution of H_3_PO_4_:H_2_O_2_:H_2_O (1:1:38). Ti/Pt/Au (10 nm/20 nm/300 nm) films were then deposited on the p^+^-InGaAs contact-ring via E-beam evaporation and photolithographic lift-off processing. The samples were then subjected to annealing in a rapid thermal annealing (RTA) chamber at 420 °C for 1 min to ensure the formation of a good p-ohmic contact [23]. Bromine-based isotropic wet etching solution (H_3_PO_4_:HBr:K_2_Cr_2_O_3_; 1:1:1) was used to define a primary circular mesa active region (40-μm diameter) as far as the n^+^-InAlAs contact layer as well as a secondary circular mesa active region (45-μm diameter) as far as the semi-insulating InP substrate to ensure device isolation [24]. The etched sample was then immersed in hot H_2_SO_4_ solution (60 °C) for 60 s followed by HF:H_2_O (1:5) treatment for 60 s in order to remove native oxides from the smooth mesa surface. Sulfur passivation was performed immediately after, by immersing the samples in (NH_4_)_2_S_x_ solution for 40–60 min in a constant temperature bath [25,26,27]. Photosensitive polyimide (CRC 8000 Series Product, Sumitomo Bakelite Co., Ltd.; Tokyo, Japan) was applied via spin-on coating and patterned for use as a capping layer over the sulfur passivated surface [28]. The polyimide was cured by ramping up the temperature (5 °C per min) until it reached 350 °C, where it was held for 30 min before being ramped down (5 °C per min) to room temperature. The entire curing process was performed under ambient N_2_. After curing, the thickness of the polyimide was approximately 4.0 µm. Polyimide within the region of the n^+^-InAlAs contact-ring was removed for n-electrode metallization. A N-ohmic contact was created by evaporating AuGeNi/Au (50 nm/500 nm) films followed by annealing in an RTA chamber at 350 °C for 30 s under ambient N_2_ to ensure a good ohmic contact. Note that a smooth mesa is essential for good surface passivation effects. Finally, SiNx was deposited to a thickness of 180 nm on the front surface of the APD as an antireflective coating. Figure 1a presents a schematic illustration of the proposed SAGFM APD. Figure 1b presents a top-view schematic diagram of the test pads on APD chips for the microwave probe used to characterize the frequency and time response. Note that the metal bond pads (Ti/Pt/Au; 20 nm/30 nm/1000 nm) were connected to the p- and n-electrodes. We sought to minimize the length of connection metallization in order to reduce the inductance component and thereby achieve high-frequency response. The performance of the fabricated APD was characterized in terms of dark current-voltage (I-V), capacitance-voltage (C-V), photo I-V, multiplication gain (M), time and frequency response.

### 2.3. Electric Field Profile Calculation

**Figure 2:**
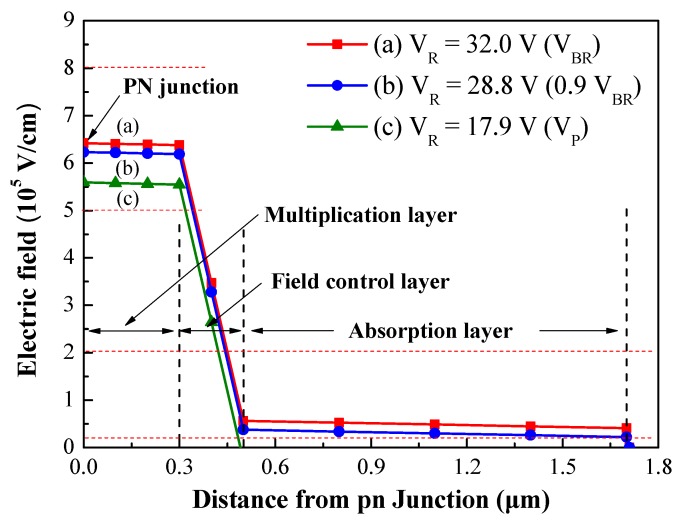
Electric field profile, calculated as a function of the distance from the p-n junction under various reverse bias voltages.

In this study, the InAlAs/InGaAs APD, light absorption and carrier multiplication processes were kept separate by employing an InGaAs absorption layer with a small band gap (E_g_ = 0.75 eV) and a InAlAs multiplication layer with a large band gap (E_g_ = 1.46 eV). Two InGaAlAs graded layers were used to shift the band gap from 0.75 eV (InGaAs) to 1.35 eV (InP) and from 0.75 eV (InGaAs) to 1.46 eV (InAlAs) to assist in the transport of carriers (generated in the absorption layer) into the multiplication and window layers. The function of the p-InAlAs field control layer was to maintain a high electric field for the multiplication layer and a low electric field for the absorption layer in order to prevent high-field induced current tunneling. The InAlAs multiplication layer requires an electric field intensity of >5 × 10^5^ V/cm to achieve carrier multiplication via impact ionization and >2 × 10^4^ V/cm to rapidly sweep out the carriers generated in the InGaAs absorption layer (i.e., prior to recombination). This requires the optimization of electric field distribution in the absorption and multiplication layers. Typically, a SAGFM APD structure would require an electric field distribution of 5 × 10^5^–8 × 10^5^ V/cm in the InAlAs multiplication layer and 2 × 10^4^–2 × 10^5^ V/cm in the InGaAs absorption layer. Figure 2 presents the electric field profile, the calculation of which was based on the parameters of the epitaxial layer in the SAGFM APD (see Table 1), as a function of the distance from the p-n junction under various reverse bias voltages. Our results indicate that the electric field intensity profile was well controlled to the range required for the InAlAs multiplication layer (5 × 10^5^–8 × 10^5^ V/cm) and the InGaAs absorption layer (2 × 10^4^–2 × 10^5^ V/cm) when the APD was operated at a voltage of 0.9 V_BR_.

## 3. Results and Discussion

### 3.1. DC Characteristics of SAGFM APD

**Figure 3:**
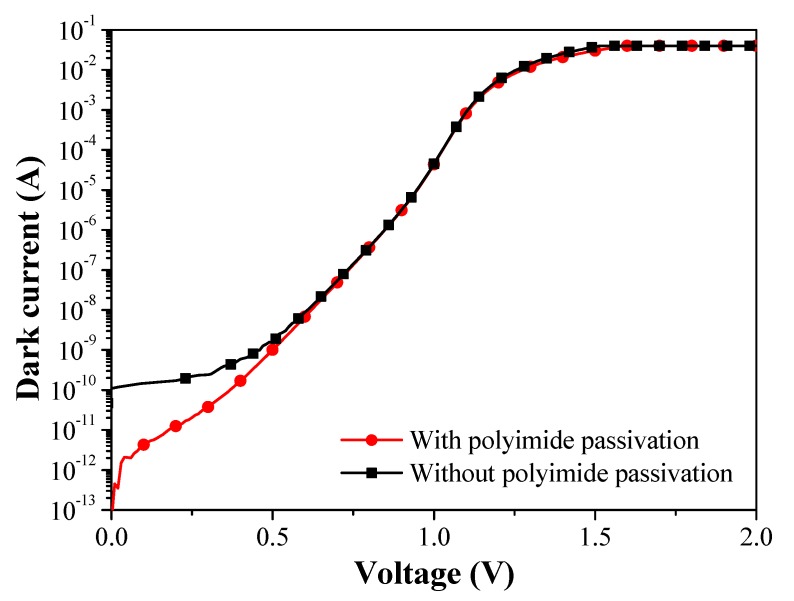
Dark I-V curves of SAGFM avalanche photodiodes (APD) before and after passivation/polyimide coating.

Figure 3 presents dark forward I-V curves obtained from the proposed SAGFM APD before and after passivation/polyimide coating. The ideality factor (n) and reverse saturation current (J_0_) of the InGaAs/InAlAs SAGFM APD (as extracted from dark I-V curves) were as follows: Before passivation/coating (n = 2.79 and J_0_ = 3.21 × 10^−7^ A/cm^2^) and after passivation/coating (n = 1.86 and J_0_ = 3.55 × 10^−9^ A/cm^2^). The reduction in n and J_0_ values after passivation are indications of suppressed surface recombination and current leakage. The series resistance (R_S_) and shunt resistance (R_SH_) of the SAGFM APD were as follows: Before passivation/coating (R_S_ = 11.94 Ω and R_SH_ = 29.7 MΩ) and after passivation/coating (R_S_ = 10.11 Ω and R_SH_ = 34.3 MΩ). The increase in R_SH_ demonstrates the effectiveness of sulfur and polyimide passivation when applied to a mesa-structure SAGFM APD. All diode parameters of the SAGFM APD are listed in Table 2.

**Figure 4:**
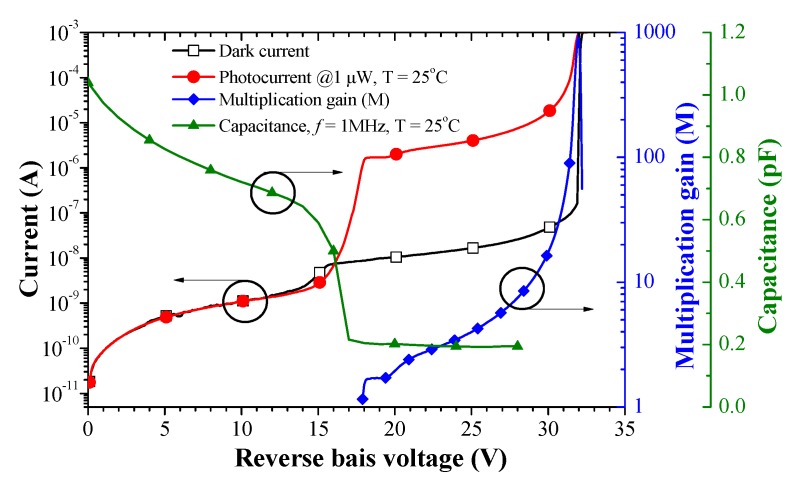
Dark current, capacitance, photocurrent and multiplication factor of InGaAs/InAlAs SAGFM APD as a function of reverse bias voltage.

Figure 4 presents the dark current (*I_D_*), capacitance (C), photocurrent (*I_ph_*) and multiplication gain (M) as a function of reverse bias voltage. Photocurrent measurements were obtained under illumination using a light source with wavelength of 1550 nm and optical power of 1 μW. The punch through voltage and the breakdown voltage of SAGFM APD were respectively 17.9 V and 32.0 V (*I_D_* of 10 μA), as measured at room temperature. At 0.9 V_BR_, the dark current was 31.4 nA, the capacitance was 0.19 pF and the multiplication gain was 9.8. At a temperature of 300 K under 1 μW illumination, the responsivity and a multiplication gain were as follows: 0.9 V_BR_ (8.04 A/W and 9.8) and 0.95 V_BR_ (18.94 A/W and 23.1). The maximum multiplication gain at V_BR_ was 994. The low dark current, low capacitance and high multiplication gain of the InGaAs/InAlAs SAGFM APD demonstrate the applicability of the proposed device for high-speed optical communication systems. For the sake of clarity, we calculated the multiplication gain (M) of the APD as follows:

(1) $$M=\frac{I_{ph}-I_{D}}{I_{pho}-I_{Do}}$$

where *I_ph_* is the APD photocurrent that has been multiplied, *I_pho_* is the unmultiplied photocurrent, *I_D_* is the APD dark current that has been multiplied and *I_Do_* is the unmultiplied dark current.

**Figure 5:**
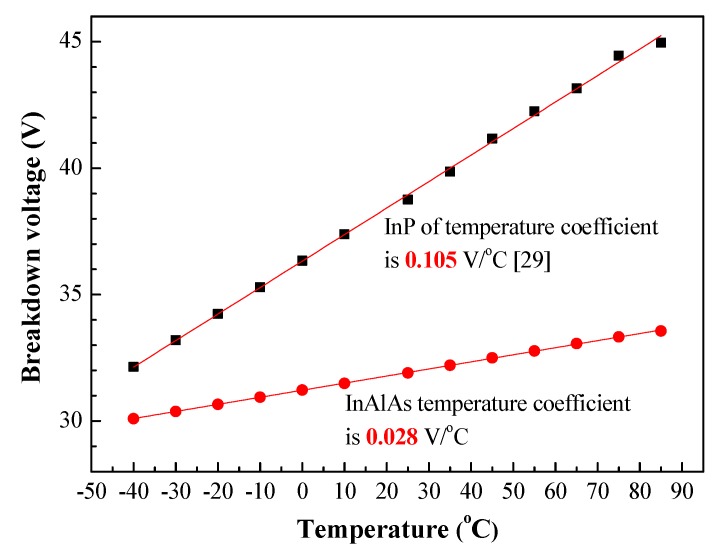
Breakdown voltage of InP-based [29] and InAlAs-based APD (current work) as a function of temperature.

Figure 5 presents the breakdown voltage of InP-based [29] and InAlAs-based APDs (current work) as a function of operating temperature. Generally, the bias of an APD is controlled with the aim of preserving the gain of the device despite fluctuations in temperature. However, most APD devices used in optical fiber communication systems are meant to be operated at temperatures between −40 °C and 85 °C. Thus, the knowledge of how avalanche breakdown voltage changes with temperature could be useful. As shown in Figure 5, the breakdown voltage temperature coefficient (ΔV_BR_/ΔT) of InP-based and InAlAs-based APDs were 0.105 V/°C [29] and 0.028 V/°C, respectively. These results demonstrate that the InAlAs-based APD was less sensitive than the InP-based APD to variations in temperature.

**Figure 6:**
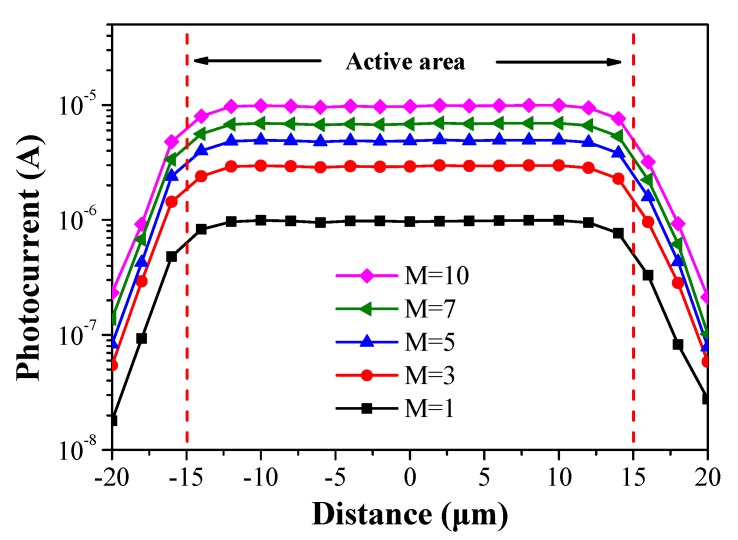
Photocurrent as a function of distance across the active area of the proposed SAGFM APD (i.e., diameter).

Figure 6 displays the photocurrent as a function of distance across the active area of the proposed mesa-structure SAGFM APD (diameter of 30 μm). Incident light was delivered using a distributed feedback (DFB) laser via a lens fiber at a wavelength of 1550 nm and a power of 1 μW. The light spot was moved in steps of 2 μm. None of the photocurrent curves presented a significant spike at the edge of the active region; however, the level of photocurrent produced within the active region was uniform, when the multiplication gain was varied between 1 and 10. This demonstrates the efficacy of the proposed mesa structure in suppressing edge-breakdown. It also demonstrates the high uniformity of the layer structure grown by MBE in terms of thickness and doping concentration in the field-control and multiplication layers.

**Figure 7:**
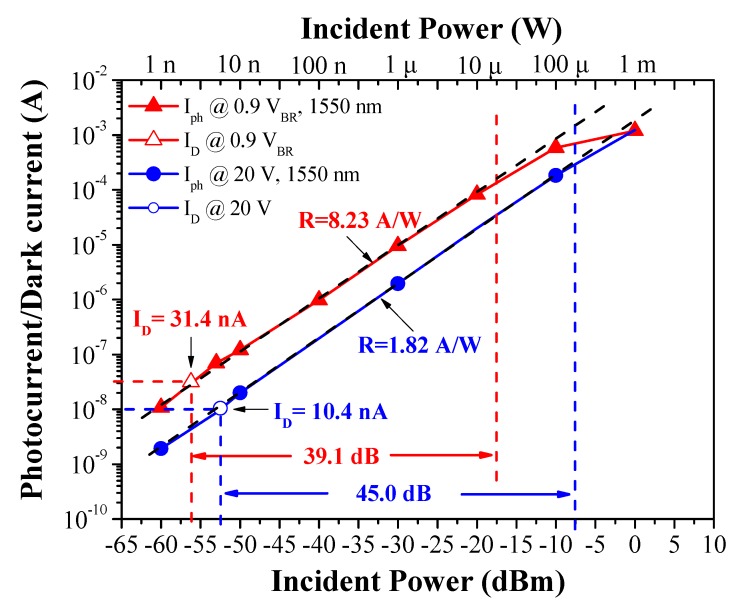
Measured photocurrent as a function of incident optical power and reverse bias voltage.

Figure 7 presents the measured photocurrent as a function of incident optical power and reverse bias voltage. Incident light was delivered using a DFB laser via a lens fiber at a wavelength of 1550 nm. The black dashed lines in Figure 7 refer to APD devices operating with constant responsivity (R) across the full range of incident optical power. Here, the responsivity of 1.82 A/W and 8.23 A/W respectively refer to InGaAs/InAlAs SAGFM APDs operated at 20 V and 0.9 V_BR_ linearly across the entire range of incident optical power. Ideally, the photocurrent values should closely match the dashed line, which would indicate that the incident optical power received by the APD covered a wide dynamic range (DR). In this study, the dynamic range of the APD device biased at 20 V (45.0 dB) was larger than that of the device biased at 0.9 V_BR_ (39.08 dB). Under high incident optical power and high voltage biasing, the multiplication factor and photocurrent generated by the APD device were both high. The internal series resistance and external load resistance associated with high output photocurrent resulted in voltage potential on the two output terminals of APD device. This voltage potential would induce an increase in the internal shunt current with the result that the output photocurrent would be saturated by the incident optical power. In contrast, under low incident optical power, the measured output photocurrent would be limited by dark current. For the sake of clarity, we calculated the dynamic range (DR) values as follows:

(2) $$DR\;\left(\mathrm{dB}\right)=10\;\log\frac{P_{H}}{P_{L}}.$$

where *P_H_* is the highest incident optical power (i.e., when output photocurrent is beginning to be saturated), *P_L_* is the lowest incident optical power (i.e., when the output photocurrent is equal to the dark current). Table 3 lists the calculated dynamic range of incident optical power.

**Figure 8:**
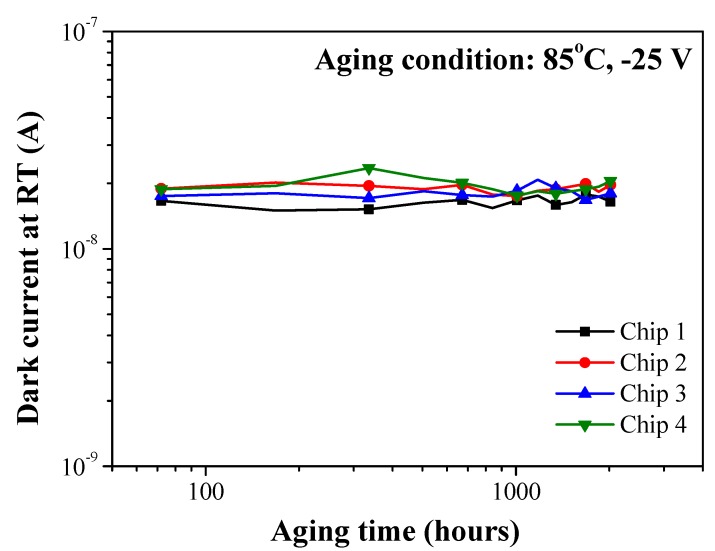
Variations in dark current measured at RT after aging. Aging conditions included reverse dark current of 100 µA at 85 °C in air atmosphere.

Figure 8 presents the reverse dark current of four SAGFM APDs as a function of aging time. Current leakage and reliability are important issues when dealing with mesa-structure APD devices. Before conducting aging tests, we first screened test samples to obtain reference data for comparisons. The aging conditions included stress reverse current of 100 μA and stress temperature of 85 °C in an air atmosphere. As shown in Table 4, testing began after approximately 100–200 h. The samples were measured at room temperature to monitor the dark current at −25 V and then returned to the aging system to repeat the procedure. We did not detect a significant variation in dark current after aging for 1344 h (i.e., 15–20 nA with S.D. <1.6 nA, which is nearly identical to the values obtained in the screening stage). These results clearly demonstrate the efficacy of the proposed sulfur/polyimide passivation scheme for the treatment of SAGFM APD with a mesa-structure.

### 3.2. AC Characteristics of SAGFM APD

**Figure 9:**
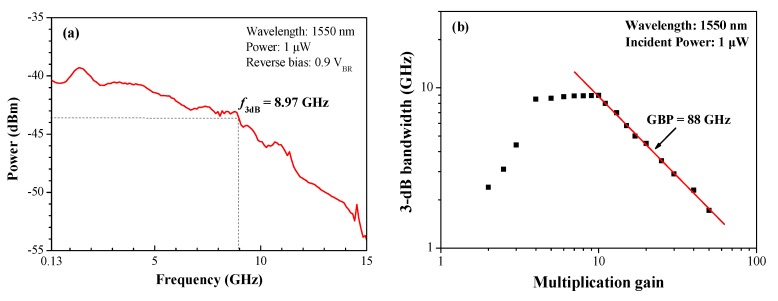
(**a**) Measured frequency response of SAGFM APD under incident optical power of 1.0 μW and reverse bias voltage of 0.9 V_BR_; (**b**) Measured *f*_3-dB_ bandwidth as a function of multiplication factor from 2 to 50.

Figure 9a presents the measured frequency response (*f*_3-dB_) of the SAGFM APD under incident optical power of 1.0 μW and reverse bias voltage of 0.9 V_BR_, as measured under load resistance of 50 Ω using a HP 8703A lightwave component analyzer. We measured *f*_3-dB_ of 8.97 GHz at the chip level, which corresponds to an operating bit rate of >10 Gbps. Figure 9b presents *f*_3-dB_ as a function of multiplication gain from 2 to 50. The highest *f*_3-dB_ bandwidth (approximately 9 GHz) was obtained within a multiplication gain range of 5–10. The *f*_3-dB_ bandwidth increased with multiplication gain between 2 and 4, due to a reduction in capacitance associated with an increase in the depletion width of the APD under reverse bias voltage. The *f*_3-dB_ bandwidth also decreased when multiplication gain exceeded 10, due to an increase in avalanche build-up time with an increase in multiplication gain. The avalanche build-up time constant increased linearly with the multiplication gain and is also proportional to the ionization coefficient ratio (k) [30]. In general, the gain-bandwidth (GB) product of APD is inversely proportional to the mean drift time of carriers within the avalanche region and is proportional to the ionization coefficient ratio [31]. Therefore, the k-ratio should be as small as possible to improve the GB-product. Overall, the gain-bandwidth product of the proposed SAGFM APD reached 88 GHz.

**Figure 10:**
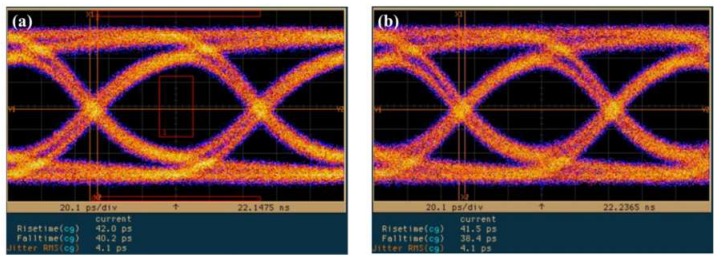

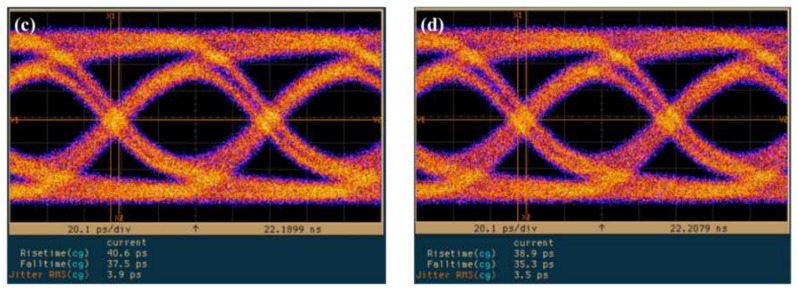
Eye diagrams of SAGFM-APD operated under a multiplication gain of 10 and at bit rates of (**a**) 10, (**b**) 11, (**c**) 12 and (**d**) 12.5 Gb/s.

**Figure 11:**
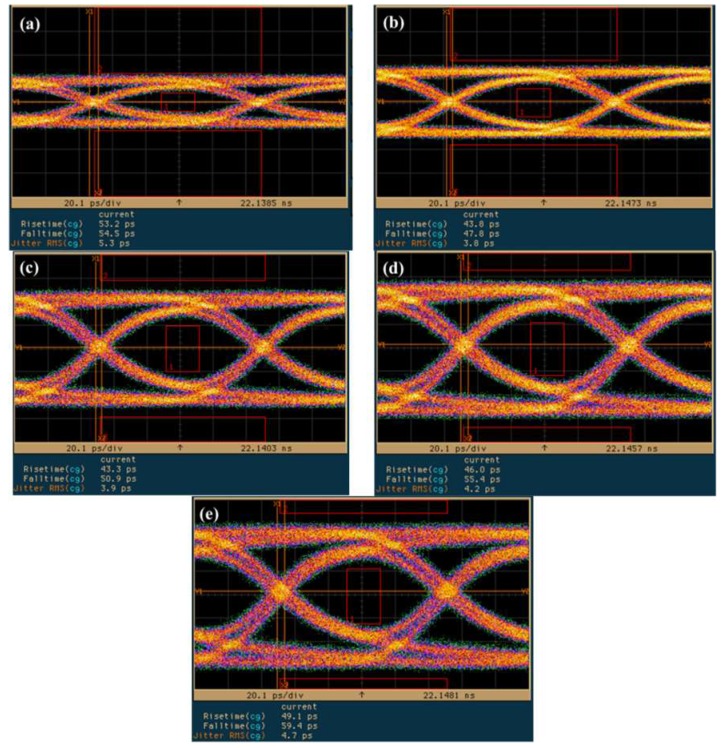
Eye diagrams of SAGFM-APD operated under multiplication gains of (**a**) 3, (**b**) 5, (**c**) 10, (**d**) 20, (**e**) 30 at a bit rate of 10 Gb/s.

**Figure 12:**
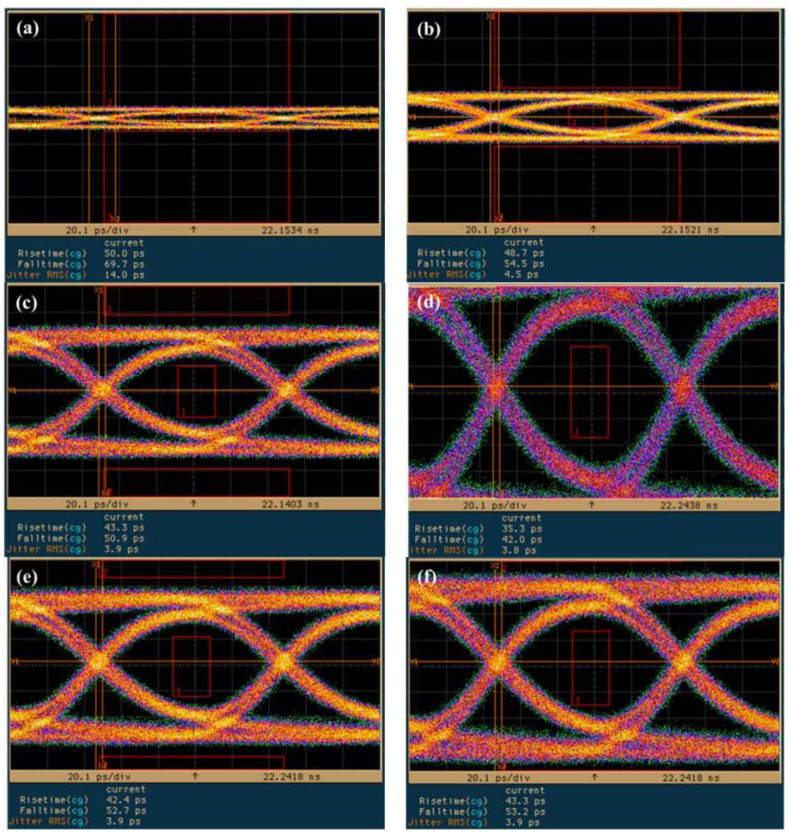
Eye diagrams of SAGFM-APD operated under incident powers of (**a**) −17.22 dBm, (**b**) −12.22 dBm, (**c**) −7.22 dBm, (**d**) −0.22 dBm, (**e**) 0.78 dBm at a bit rate of 10 Gb/s with a multiplication gain of 10. Note: (**a**–**d**) The vertical scale is 15 mV; (**e**,**f**) the vertical scale is 40 mV.

Figure 10 presents eye diagrams of the InGaAs/InAlAs SAGFM APD, under a fixed multiplication gain of 10 using nonreturn-to-zero (NRZ) pseudorandom codes with a length of 2^31^-1, under operating speeds corresponding to bit rates of (a) 10, (b) 11, (c) 12 and (d) 12.5 Gb/s. A rise time of approximately 38.9–42.0 ps was derived from the eye-diagrams. Observe that we detected a notable absence of intersymbol-interference and the signals remained error-free at data-rates of up to 12.5 Gbps. Figure 11 presents the eye diagrams obtained from the InGaAs/InAlAs SAGFM APD operated under multiplication gains of (a) 3, (b) 5, (c) 10, (d) 20, (e) 30 with the bit rate fixed at 10 Gb/s. A 10 Gb/s built-in eye mask was used to evaluate the APD operated with a suitable multiplication gain; i.e., where the transmitted signals would be not present within the eye mask region. We determined that multiplication gains of 5 and 10 were the optimal choices. We sought to characterize the dynamic range of the AC optical power by obtaining eye diagrams of InGaAs/InAlAs SAGFM APD operated at 10 Gb/s with various incident optical powers (−17.22 dBm, −12.22 dBm, −7.22 dBm, −0.22 dBm and 0.78 dBm) at room temperature, as shown in Figure 12. Similarly, the transmitted signals should not be present within the eye mask region. This was achieved when the dynamic range of incident optical power ranged from −12.22 dBm to 0.78 dBm. Thus, the dynamic range of AC incident optical power was approximately 13 dB, which is less than that of DC incident optical power (45 dB). Table 5 lists time response and calculation of *f*_3-dB_ data using values obtained from eye diagram measurements.

## 4. Conclusions

This paper presents the fabrication and characterization of InAlAs/InGaAs SAGFM APDs with a mesa-structure to enable high-speed operations. The side walls of the mesa underwent sulfur treatment before being coated with a thick polyimide passivation layer, to reduce current leakage, reduce capacitance, increase response speeds and enhance reliability. The passivation effects were shown to reduce surface recombination and current leakage, as indicated by a reduction in ideality factor and reverse saturation current. The breakdown temperature coefficient of InGaAs/InAlAs APD was approximately 0.028 V/°C, which indicates that the proposed device is highly insensitive to temperature variations. Under 0.9 breakdown voltage biasing, we obtained impressive results in terms of dark current (31.4 nA), capacitance (0.19 pF) and multiplication gain (10) at room temperature. The dynamic range of DC incident optical power was approximately 45 dB, which is higher than that of AC incident optical power (13 dB). We observed only a negligible change in dark current (i.e., 15–20 nA) after aging for 1344 h. We also obtained *f*_3-dB_ of 8.91 GHz and gain-bandwidth product up to 88 GHz. In eye-diagrams, there was also a notable absence of intersymbol-interference and the signals remained error-free at data-rates of up to 12.5 Gbps.

## Figures and Tables

**Table 1:** Details of epitaxial layers in SAGFM APD.

Layer Name	Epitaxial Layer	Thickness (µm)	Concentration (cm^−3^)
Contact	P^+^-InGaAs	0.1	1.0–3.0 × 10^19^
Window	p-InP	0.35	5.0–6.0 × 10^18^
Grading	i-InGaAlAs	0.03	Undoped
Absorber	i-InGaAs	1.2	Undoped
Grading	i-InGaAlAs	0.03	Undoped
Field control	p-InAlAs	0.2	2.0 × 10^17^
Multiplication	i-InAlAs	0.3	Undoped
Contact	n^+^-InAlAs	1.0	5.0–6.0 × 10^18^
Buffer	n-InP	0.5	1.0–5.0 × 10^17^
Semi-insulating	--	350	--

**Table 2:** Diode performance parameters of SAGFM APD.

Parameter	Symbol	With Polyimide	Without Polyimide	Unit
Series resistance	Rs	10.11	11.94	Ω
Shunt resistance	Rsh	34.3	29.7	MΩ
Ideality factor	n	1.86	2.79	-
Reverse Saturation Current Density	J_0_	3.55 × 10^−9^	3.21 × 10^−7^	A/cm^2^

**Table 3:** Dynamic range of incident optical power.

	DR (dB)	P_H_ (W)	P_L_ (W)
@0.9 V_BR_	39.08	1.78 × 10^−5^	2.2 × 10^−9^
@20 V	45.02	1.78 × 10^−4^	5.6 × 10^−9^

**Table 4:** Variations in the dark current of SAGFM APDs after stressed aging.

Aging Time (hours)	Chip 1 I_D_ (nA)	Chip 2 I_D_ (nA)	Chip 3 I_D_ (nA)	Chip 4 I_D_ (nA)
0	15.5	17.9	16.8	17.6
72	16.6	18.9	17.5	18.8
168	15.0	20.2	18.0	19.5
336	15.2	19.5	17.1	23.5
504	16.3	18.8	18.4	21.2
672	16.8	19.7	17.7	20.1
840	15.4	17.8	17.4	18.8
1008	16.7	17.5	18.5	17.6
1176	17.6	18.5	20.8	18.4
1344	15.9	18.8	19.0	17.9
1512	16.4	19.4	18.4	18.4
1680	17.8	19.9	16.8	18.8
1848	17.4	18.3	17.4	19.3
2016	16.5	19.7	18.0	20.5
Standard Deviation (S.D.; nA)	0.8	0.8	1.0	1.6

**Table 5:** Time response and calculation of *f*_3-dB_ data using values obtained from eye diagram measurements.

Speeds @M = 10	Rise Time (ps)	Calculate *f*_3-dB_ (GHz)
Bit rate: 10 Gb/s	42.0	8.33
Bit rate: 11 Gb/s	41.5	8.43
Bit rate: 12 Gb/s	40.6	8.62
Bit rate: 12.5 Gb/s	38.9	8.99
Multiplication Gain @10 Gb/s	--	--
M = 3	53.2	6.58
M = 5	43.8	7.99
M = 10	43.3	8.08
M = 20	46.0	7.61
M = 30	49.1	7.13
Incident power (dBm) @10 Gb/s, M = 10	--	--
−17.22	50.0	7.00
−12.22	48.7	7.19
−7.22	43.3	8.08
−2.22	35.3	9.91
−0.22	42.4	8.25
0.78	43.3	8.08
